# Supplementary material for: Time‑resolved multi-omic analysis of paclitaxel exposure in human iPSC‑derived sensory neurons unveils mechanisms of chemotherapy‑induced peripheral neuropathy
Source: Cell Death Dis. 2026 Feb 10;17(1):211. doi: 10.1038/s41419-026-08445-2 (PMC12921266; doi:10.1038/s41419-026-08445-2)
Supplement: Supplementary file 15 — Dataset 1. [file 41419_2026_8445_MOESM15_ESM.zip › CDD_Open_Data/04_RNA_Seq/Timeline_panel_plots_etc_2023-01-23_1609.zip!/Panel_Plots_etc/Evidence_Plots/48h/evidence_plot_standard contrasts_msigdb_reactome_48h_PTX_vs_DMSO_ID5_M9648_REACTOME_FORMATION_OF_TUBULIN_FOLDING_INTERMEDIATES_BY_CCT_TRIC.pdf]

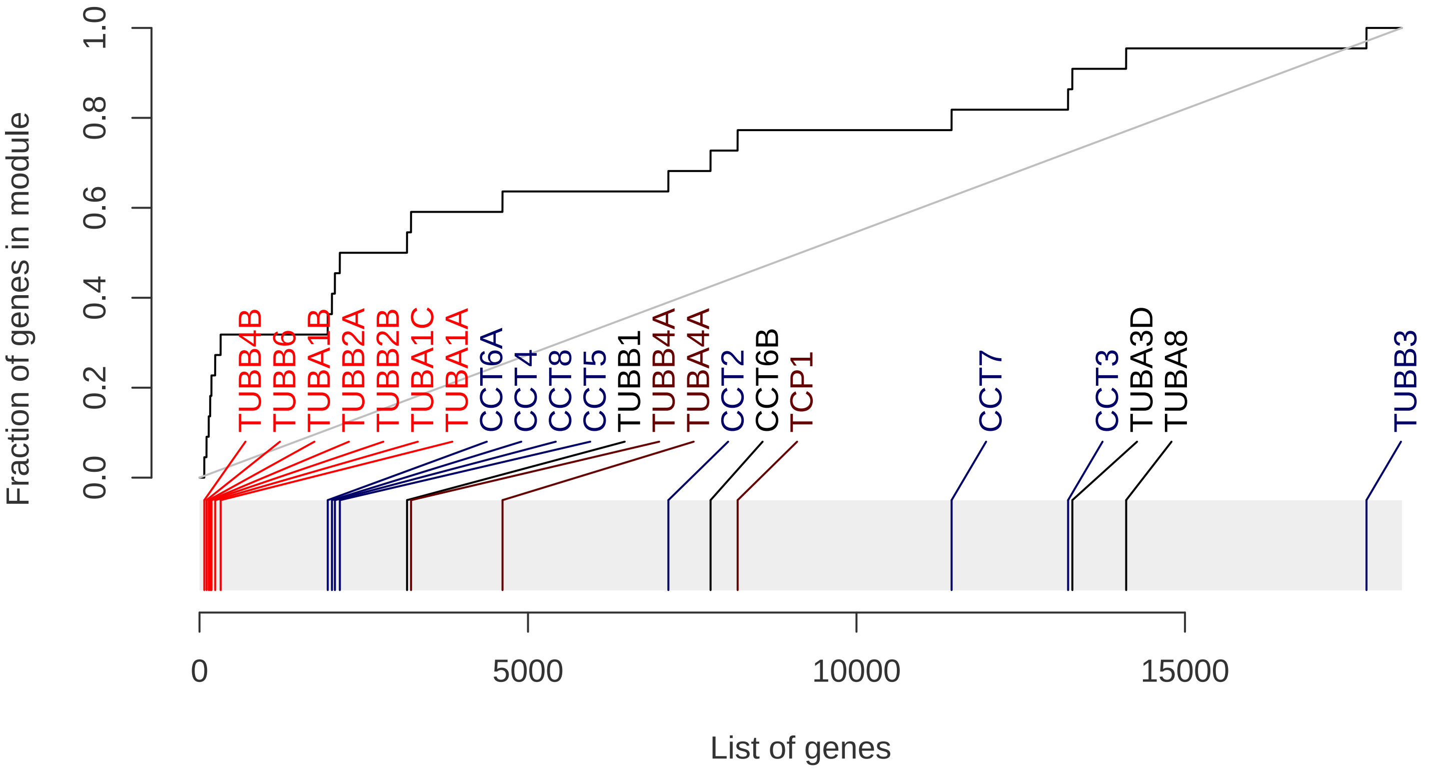

Fraction of genes in module
0.0
0.2
0.4
0.6
0.8
1.0
TUBB4B
TUBB6
TUBA1B
TUBB2A
TUBB2B
TUBA1C
TUBA1A
CCT6A
CCT4
CCT8
CCT5
TUBB1
TUBB4A
TUBA4A
CCT2
CCT6B
TCP1
CCT7
CCT3
TUBA3D
TUBA8
TUBB3
0
5000
10000
15000
List of genes
